# Investigation of the Compressibility and Compactibility of Titanate Nanotube-API Composites

**DOI:** 10.3390/ma11122582

**Published:** 2018-12-18

**Authors:** Barbara Sipos, Klára Pintye-Hódi, Géza Regdon, Zoltán Kónya, Maryléne Viana, Tamás Sovány

**Affiliations:** 1 Institute of Pharmaceutical Technology and Regulatory Affairs, University of Szeged, Eötvös u. 6., H-6720 Szeged, Hungary; sipi08@gmail.com (B.S.); klara.hodi@pharm.u-szeged.hu (K.P.-H.); geza.regdon@pharm.u-szeged.hu (G.R.J.); 2 Department of Applied and Environmental Chemistry, University of Szeged, Rerrich Béla tér 1., H-6720 Szeged, Hungary; konya@chem.u-szeged.hu; 3 Reaction Kinetics and Surface Chemistry Research Group, Hungarian Academy of Sciences-University of Szeged, Rerrich Béla tér 1, H-6720 Szeged, Hungary; 4 Department of Pharmaceutical Technology, Faculty of Pharmacy, University of Limoges, LCSN EA 1069, 87000 Limoges, France; marylene.viana@unilim.fr

**Keywords:** titanate nanotube, diltiazem hydrochloride, diclofenac sodium, atenolol, hydrochlorothiazide, composite, compressibility

## Abstract

The present work aims to reveal the pharma-industrial benefits of the use of hydrothermally synthesised titanate nanotube (TNT) carriers in the manufacturing of nano-sized active pharmaceutical ingredients (APIs). Based on this purpose, the compressibility and compactibility of various APIs (diltiazem hydrochloride, diclofenac sodium, atenolol and hydrochlorothiazide) and their 1:1 composites formed with TNTs were investigated in a comparative study, using a Lloyd 6000R uniaxial press instrumented with a force gauge and a linear variable differential transformer extensometer. The tablet compression was performed without the use of any excipients, thus providing the precise energetic characterisation of the materials’ behaviour under pressure. In addition to the powder functionality test, the post-compressional properties of the tablets were also determined and evaluated. The results of the energetic analysis demonstrated that the use of TNTs as drug carriers is beneficial in every step of the tabletting process: besides providing better flowability and more favourable particle rearrangement, it highly decreases the elastic recovery of the APIs and results in ideal plastic deformation. Moreover, the post-compressional properties of the TNT–API composites were found to be exceptional (e.g., great tablet hardness and tensile strength), affirming the above results and proving the potential in the use of TNT carriers for drug manufacturing.

## 1. Introduction

Titanate nanotubes (TNTs) are recently explored nanomaterials with special physicochemical properties and versatile applicability, from engineering [1,2,3,4,5] to therapeutic use [6,7,8,9,10]. 

Based on the synthesis method, two types of TNTs can be distinguished, anodized and hydrothermally synthesised, of which both have a particular tubular shape and show good physicochemical stability [11,12]. Focusing on pharmaceutical applications, these characteristics indicate that TNTs can be filled with drugs, and may serve as drug carriers for therapeutic use. In this regard, anodized TNTs, which are surface attached carriers, may beneficially be used in implantology [13,14,15], while hydrothermally synthesised TNTs, which are separate carriers, can be loaded and/or functionalized with active substances [16,17] and provide a large spectra of drug delivery approaches. Concentrating on hydrothermally synthesised TNTs, it is important to underline that these nano-materials are completely biocompatible and assure safe use without any toxicity risks, as discussed in several studies [18,19]. This property makes them much more valuable for nanomedicine than many other nano-materials, e.g., carbon nanotubes, and necessitates the detailed pharmaceutical investigation of TNTs.

In our previous study [20], hydrothermally synthesised TNTs were characterised based on their ability to be incorporated with active pharmaceutical ingredients (API). A 1:1 weight ratio of API–hydrothermally synthesised titanate nanotube (API–TNT) composites were prepared and investigated in terms of morphology, surface free energy, thermal properties, API–TNT bonds and drug release. In general, composite formation may bear numerous advantages, such as improved mechanical properties, better stability, electrochemical properties or biocompatibility [21,22]. Based on this, our hypothesis was that the composite formation may stabilize the tested APIs in nanocrystalline form, improve their processability, prevent auto-aggregation and therefore improve the dissolution profile of the APIs. It was seen that TNTs were suitable to be loaded with drugs, however, the efficacy of API incorporation depended on the API characteristics, e.g., diltiazem hydrochloride and diclofenac sodium could completely fill the TNTs, while the incorporation of atenolol and hydrochlorothiazide was only partially achievable. Based on the results, hydrogen bonds were the main interactions between the APIs and the TNTs which, together with other findings, proves that the efficacy of the composite formation, and the strength of association, were mainly defined by the hydrogen donor capacity of the incorporated APIs. In the case of important association, as seen for diclofenac sodium–TNT composites, the composite formation was shown to affect the drug dissolution and result in modified drug release, which suggests the implementation of composite containing modified-release tablets.

Based on the characteristics of TNTs, and the fact that the tablet is still the most popular dosage form on the patients’ part and is also the most frequently chosen form by industry, it is truly worth focusing on the innovative tabletting solutions titanate nanotubes may provide. First of all, a great advantage of API–TNT composites is that the API is kept in a nano-sized form inside the tubes, and is therefore protected from autoaggregation. As a result, TNTs offer the opportunity of improving the solubility of the incorporated API, thereby leading to better bioavailability. At the same time, composite formation may offer the realisation of controlled drug release, due to the position of the API inside the tube and the bonds forming between the APIs and the TNTs as mentioned above. Besides these approaches, another important aspect of the composites is worth pointing out: while the manufacturing of an API requires precise knowledge about powder properties and requires detailed investigations, API–TNT composites may ease and shorten the preformulation studies and make the tabletting process easily predictable, since the composites presumably will behave similarly in spite of carrying different APIs.

With the purpose of confirming the statement described above, the present study aimed to investigate the compressibility and compactibility of API–TNT composites, and reveal the advantages they can bring. In order to be able to draw useful and correct conclusions, four different APIs and their composites formed with TNTs were examined and compared regarding their mechanical properties. The powder compression mechanisms of materials are usually estimated by fitting data into mathematical (Heckel [23,24], Walker [25] and Kawakita-Lüdde [26]) models, which are widely used in the field of pharmaceutical technology [27,28,29]. However, it is notable that, besides some critical issues [30], these models describe only a given part of the compression cycle, and need to be combined with each other to give a complete view of the process. Moreover, these equations are based on the volume/density reduction of the powder bed, which may hide some important parts of the compression process, especially if they are calculated according to the out-die method. Accordingly, we decided to apply a less known but certainly more detailed and complex method to reveal the energetic background behind the behaviour of TNT based nanomaterials under compression pressure. The measurements of the present study were therefore performed with a Lloyd 6000R uniaxial press instrumented with a force gauge and a linear variable differential transformer extensometer, which allowed thorough analysis of the whole compression cycle of the pure materials. Moreover, the post-compressional properties of the tablets could also be determined with the same equipment and taken into consideration. The suitability of this methodology has already been proven by the work of Viana et al. in comparing the compression performance of different types of celluloses [29]. The present work attempted to test the powder functionality of various API–TNT composites, and thereby investigate a completely unexplored but quite reasonable aspect of these composites, which may open new directions in the tabletting of nano-sized APIs.

## 2. Materials

**Figure 1:**
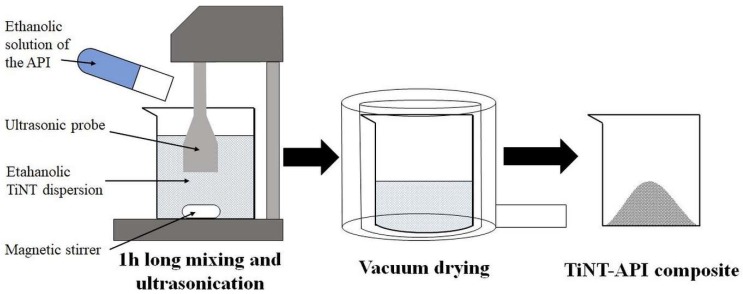
Schematic representation of the composite formation process.

**Figure 2:**
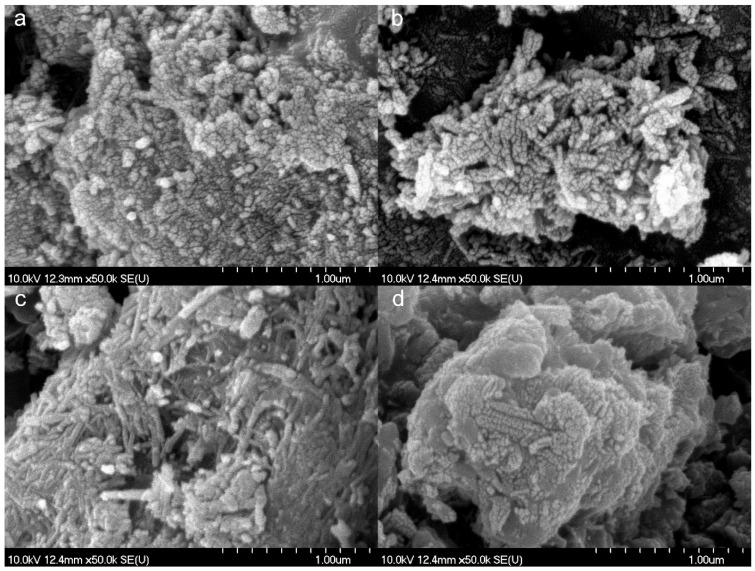
Scanning electron micrographs of the diltiazem hydrochloride (DiltTi) (**a**), diclofenac sodium (DicTi) (**b**), atenolol (ATNTi) (**c**) and hydrochlorothiazide (HCTTi) (**d**) composites.

Diltiazem hydrochloride (DiltHCl), diclofenac sodium (DicNa), atenolol (ATN) and hydrochlorothiazide (HCT) were kindly supplied by Sanofi-Aventis PLC (Budapest, Hungary), Egis Pharmaceuticals PLC (Budapest, Hungary), TEVA Pharmaceuticals PLC (Debrecen, Hungary and Gedeon Richter PLC (Budapest, Hungary), respectively. Hydrothermally synthesised titanate nanotubes (TNT) and 1:1 weight ratio composites of APIs and TNTs (abbreviated with DiltTi, DicTi, ATNTi and HCTTi for dilthiazem hydrochloride, diclofenac sodium, atenolol and hydrochlorothiazide containing composites, respectively) were produced by the University of Szeged, Department of Applied and Environmental Chemistry according to the method described by Sipos et al. [20]. In general, a 1:1 ratio of alcoholic API solution and the same 1:1 ratio of alcholic TNT dispersion were mixed and subjected to an hour-long ultrasonic treatment, followed by removal of solvent in a vacuum dryer (Figure 1) [20]. This method resulted in a perfect composite formation for DiltHCl and DicNa, where the API was incorporated into the tubular structure or attached to the surface of the TNTs in nanocrystalline form. In the case of HCT, a reverse composite formation was observed, where the TNTs were attached to the surface of the re-crystallising HCT, while for ATN the composite formation was unsuccessful and, due to the weakness of interaction, the composite acted like a physical mixture of the API and TNTs (Figure 2) [20].

## 3. Methods

Following the method introduced by Viana et al. [31] for powder functionality, the compressibility of the TNTs, APIs and API–TNT composites was investigated using a Lloyd 6000R uniaxial press (Ametek SAS Lloyd Inst, Elancourt, France), instrumented with a force gauge and a linear variable differential transformer extensometer. The equipment provided the tabletting of the samples without using any excipients, and therefore allowed thorough investigation of the behaviour of the components under compression pressure. The compacts were prepared in a 1 cm^3^ stainless steel cell with manual filling, using 50, 100, 150, 200 and 250 MPa pressures for each material. The weight of the powder was determined using the bulk density of the unpacked powders. A minimum of 3 tablets per compression pressure were made from each component, with a tabletting punch speed of 1 mm/sec. Force and displacement data were processed by a computer (R-Control software, Version 2.0, Lloys Inst LTD, Fareham, UK) connected to the equipment.

### 3.1. Packing Coefficient

The packing coefficient (C_t_, %) was calculated using Equation (1), on the basis of the obtained force–displacement data of at least 10 compacts per material.

C_t_ = ((H_0_−H_0.5_)/H_0_) × 100 (1)

where H_0_ is the height of the initial powder bed and H_0.5_ is the height of the powder bed at 0.5 MPa compression pressure. This value gives information about the particle rearrangement in the initial phase of the compression process, and correlates with the Compressibility Index and the Hausner Ratio. It indicates the densification behaviour of a powder at low pressure as follows: C_t_ > 30.0 % displays bad particle rearrangement, C_t_ < 25.0 % refers to optimal behaviour and a value in between indicates fair densification properties [31].

### 3.2. Energetic Analysis

**Figure 3:**
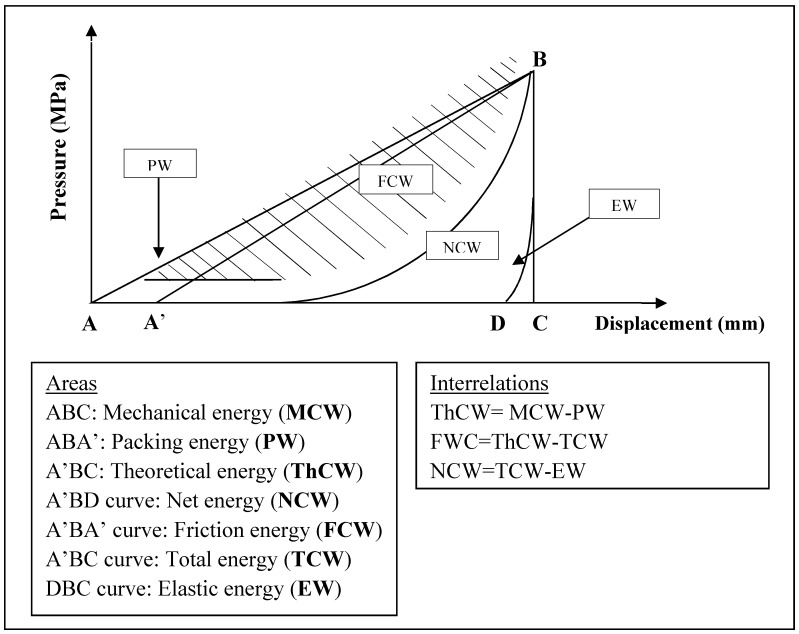
Schema of the compression cycle and the associated energies.

The energetic analysis describing the energy utilisation of the materials during the compression cycle was evaluated with Origin 7.5 software (OriginLab Corporation, Northampton, MA, USA), and aimed to determine the energies displayed in Figure 3. The total mechanical energy (MCW) involved in the compression process (ABC triangle) is composed of four partial energy segments: packaging energy (PW), friction energy (FW), net energy (NCW) and elastic energy (EW), which are used in the initial packaging of the particles, particle–particle and particle–die frictions during densification, plastic and elastic deformations respectively. With the subtraction of PW (ABA’ triangle, where A’ is the smallest displacement of punch that generates measurable force) from the MCW, the theoretical energy (ThCW) (A’BC triangle) may be calculated, which is related to the energy utilised for powder compaction. If the FW (A’BA’ curved area) is subtracted from the ThCW itself, we may calculate the total energy (TCW) (A’BC curved area) of the deformations. The TCW can be further segmented to the net energy (NCW) (A’BD curved area), utilised for plastic deformations, and elastic energy (EW) (DBC curved area), which is gained by elastic recovery of the particles.

The determined energies were normalised to compact weight, and used to estimate the linear interrelations between given energies and their change all over the applied compression pressure range [31].

The following R_i_ and their complementary R’_i_ yields signify the pressure related transformation of MCW into ThCW (R_1_) and PW (R’_1_), the ThCW into TCW (R_2_) and FWC (R’_2_), and the TCW into NCW (R_3_) and EW (R’_3_), calculated by applying Equations (2)–(7), respectively:

R_1_ = ΔThCW/ΔMCW (2)

R’_1_ = 1−(ΔThCW-ΔMCW) (3)

R_2_ = ΔTCW/ΔThCW (4)

R’_2_ = 1−(ΔTCW/ΔThCW) (5)

R_3_ = ΔNCW/ΔTCW (6)

R’_3_ = 1−(ΔNCW/ΔTCW) (7)

R_1_ is related to the packaging ability of the materials, and represents the remaining energy in percentage after the initial rearrangement of the particles in the first phase of compression. R_2_ is indicative of the frictions in the second phase of compression, while R_3_ and R’_3_ display the percentage of the plastic and elastic deformation energies of the materials, respectively. In consequence, R_3_ equals the plasticity of the materials, calculated according to Stamm and Mathis [32].

The efficacy of the conversion of net energy into cohesion during compression was investigated by the efficacy coefficient (C_eff_, %), defined as the slope of the linear part of the (BW) = f(NCW) function plot, where BW is the breaking energy discussed below. A value superior to 0.1% indicates good energy utilisation of the material [31].

A general view on the powder behaviour under pressure, showing the ability of the material to be compacted by the direct compression method, was given by the global yield (R_T_, %), which was determined by Equation (8):

R_T_ = R_1_ × R_2_ × R_3_ × C_eff_ (8)

### 3.3. Post-Compressional Properties

The determination of tablet properties can help to estimate the efficiency of the tabletting process, and thereby can be used to complete and confirm the results of the energetic measurements.

The geometric parameters of the tablets were tested 24 h after ejection by investigating 3 tablets per pressure per component. The height of the tablets was measured with a calliper, while the diameter was determined as part of the breaking test. The as measured geometric parameters served to define the compaction ratio (ρ, %) of the compacts using Equation (9):

ρ = (d_compact_/d_pycno_) × 100 (9)

where d_compact_ is the density of the tablet after ejection and d_pycno_ is the density of the initial powder determined by a pycnometer. The pycnometric density was defined using an AccuPyc 1330 pycnometer (AccuPyc 1330 series No. 2441, Micromeritics Instruments Inc., Norcross, GA, USA). Measurements were repeated until the value stabilised [33].

**Figure 4:**
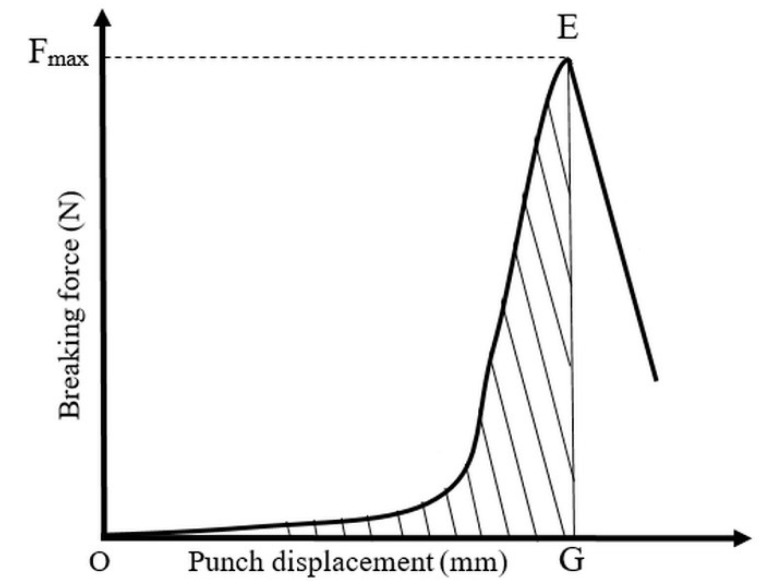
General diametral rupture curve of DiltTi compressed with 100 MPa.

The breaking test of the tablets was performed in standing tablet posture with the Lloyd 6000R uniaxial press. The measurements of breaking force and tablet diameter provided force–displacement rupture plots, which were used to determine the breaking energy and tensile strength. A typical rupture of the API–TNT composites is seen in Figure 4. The breaking energy (BW, J·g^−1^) was obtained from Equation (10):

BW = OEG/m(10)

where OEG is the area under the curve of the breaking process and m is the mass of the tablet.

The tensile strength (R_d_, MPa) of the tablets was defined by applying Equation (11):

R_d_ = 2*F_max_*/*πdh* (11)

where *F_max_* is the breaking force in N, *d* is the diameter, and *h* is the thickness in mm of the tablet after ejection.

## 4. Results and Discussion

### 4.1. Packing Coefficient

The packing coefficients (Table 1) allowed understanding and comparison of the particle rearrangement properties of the materials in the initial phase of the compression process.

It is remarkable that TNTs showed an excellent C_t_ value, far below 10.0%, despite the needle shaped crystal character, which was certainly due to the roundish agglomerates they form [20] (see Supplementary Materials) playing a driving role in the rheological properties. Moreover, the rearrangement profile of the API–TNT composites was also much more favourable than that of the pure APIs. Although the positive effect of composite formation on the packing character of the incorporated APIs was clear, the rate of the influence differed by API, resulting in the following order of C_t_ improvement: DiltHCl > DicNa > ATN > HCT. This result can be explained by the structure of the composite products presented in a previous study [20]. For DiltHCl and DicNa, where the composite formation was perfect, the properties of TNTs completely dominated the packaging properties; the different improvement rate may be due to the different agglomeration mechanisms resulting in different surface coverage of the TNTs with the API. In the case of ATN, the TNTs were intercalated between the API particles, while for HCT the moderate improvement may be due to the increase in the surface free energy and adhesivity of the composite, since here the TNTs formed the outer layer.

### 4.2. Energetic Analysis

**Figure 5:**
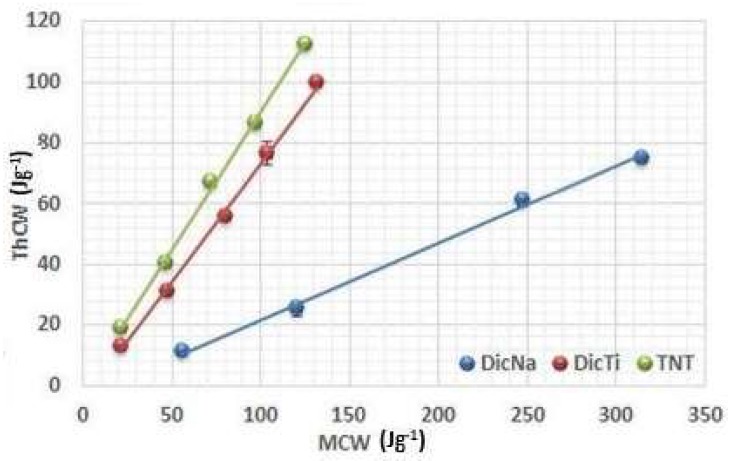
Evolution of ThCW with MCW of TNT, DicNa and DicTi at 50, 100, 150, 200 and 250 MPa compression pressure.

**Figure 6:**
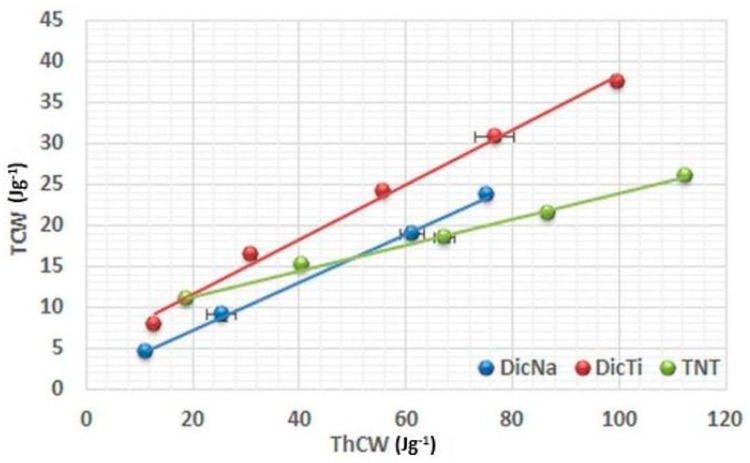
Evolution of total energy (TCW) with theoretical energy (ThCW) of TNT, DicNa and DicTi at 50, 100, 150, 200 and 250 MPa compression pressure.

**Figure 7:**
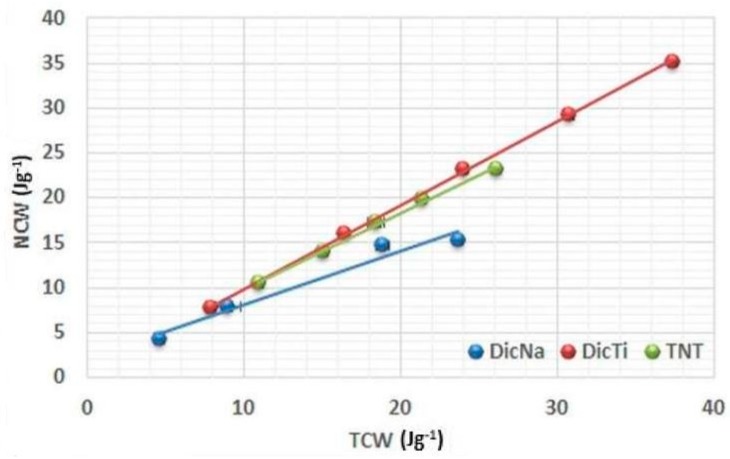
Evolution of net energy (NCW) with TCW of TNT, DicNa and DicTi at 50, 100, 150, 200 and 250 MPa compression pressure.

Figure 5, Figure 6 and Figure 7 present the plots of the energetic analysis of TNT, DicNa and DicTi products, and are good representatives of the other investigated APIs and composites as well (other curves may be seen in Supplementary Materials). It can be seen that all the curves are linear, however, outstanding differences can be seen in the slope of the curves. In order to quantify and compare these differences, R_i_ and R’_i_ values were determined from the slope of (ThCW) = f(MCW), (TCW) = f(ThCW) and (NCW) = f(TCW) plots. The results are summarised in Table 2. R’_i_ values may be calculated according to 100 − R_i_ equation.

The results obtained by the investigation of the C_t_ are well supported by the analysis of the conversion of mechanical energy into theoretical energy (Figure 5). The high slope means that only a small proportion of the applied MCW is lost on the initial particle rearrangement and converted into ThCW.

The very high R_1_ value of the pure TNTs indicates good flowability and favourable particle rearrangement. In contrast, small R_1_ values were observed for all APIs, which is related to the considerable energy loss on packaging and rearrangement of the particles due to poor flowability and high adhesivity, which made the experiments extremely hard to accomplish. As concerns the composite products, it is noteworthy that the plots of the (ThCW) = f(MCW) function of the composites display a comparable rise of curve with the pure TNTs instead of the incorporated APIs (Figure 5), supporting their good flowability and packaging ability. Nevertheless, it is important to note that the R_1_ values belonging to the composites showed the same differences in comparison with the TNTs as was observed for the packaging coefficient, and these are due to differences in the efficacy of the incorporation process.

In order to identify the energy dissipated to friction, TCW vs. ThCW plots were evaluated (Figure 6).

The low R_2_ values (Table 2) of the pure TNTs indicate an ineffective conversion of theoretical energy into total energy, due to a massive energy loss caused by friction under pressure. This may be evoked by the high surface free energy of the TNTs (80.72 mJ/m^2^) [20]. In contrast, due to their lower surface free energy the APIs displayed relatively high *R*_2_ values, indicating modest energy loss to friction during compression. Regarding the composites, it may be observed that the slope of curve is highly related to the surface coverage and consequential surface free energy of the various composites (see Supplementary Materials). For those composites where the TNTs were covered with drug nanocrystals (DicTi (Figure 6) and DiltTi), the slopes were parallel with those of the pure APIs, while the behaviour of HCTTi was predominated by the characteristics of TNTs, supporting the properties discussed before. The unexpected behaviour of ATNTi, which showed a decreased rate of friction compared to both the pure API and the TNTs, may be explained by weak hydrophilic interactions between the API and the nanotubes, resulting in more hydrophobic characteristics of this physical mixture like composite.

Plotting the (NCW) = f(TCW) function served to investigate the conversion of the total energy into net energy (Figure 7), where the high slope and the resulting R_3_ values indicate plastic deformation without considerable elastic recovery.

High R_3_ values were obtained for TNTs and composites, which indicates that the good plastic deformation of TNTs may highly improve this property of the APIs with composite formation. This was especially remarkable in the case of DicNa, where the lamination of the tablets compressed from the pure API was clearly visible above 150 MPa, indicating considerable elastic recovery after compression. Similar good results were observed for DiltHCl, but considerably lower values may be observed for the ATNTi and HCTTi composites, indicating that the rate of improvement strongly depends on the success of the composite formation process. In the case of incomplete incorporation of the API into the TNTs, the physical properties of the drug may predominate the behaviour of composites, but the improvement in the results of the strongly elastic ATN indicates that the presence of TNTs may have a positive effect on this characteristic, even if they are presented in the form of a physical mixture.

Overall, it can be concluded that composite formation highly improved the compression properties and energy utilisation during compression, due to better flowability and particle rearrangement and improved plasticity of the incorporated APIs, which presumes better post-compressional tablet properties. Interestingly, the less favourable property of the TNTs (low R_2_ value) did not dominate in the composite products, therefore no substantial negative effects of composite formation could be revealed by the measurements. However, it is noteworthy that the rate of the positive impact provided by the TNTs was considerably affected by the efficacy of the incorporation process.

### 4.3. Post-Compressional Properties

The good post-compressional properties of tablets are essential for many manufacturing and pharmacological aspects. In addition, tablet properties can also serve to estimate the efficacy of the tabletting process, which—in our case—refers to the compactibility of the given materials. Therefore, post-compressional attributes were used to confirm the results of the energetic analysis. The compaction ratios were determined from the results of the tablet geometry measurements and the previously performed pycnometric examinations, while breaking tests were used to define the tensile strength of the tablets (Table 3).

**Figure 8:**
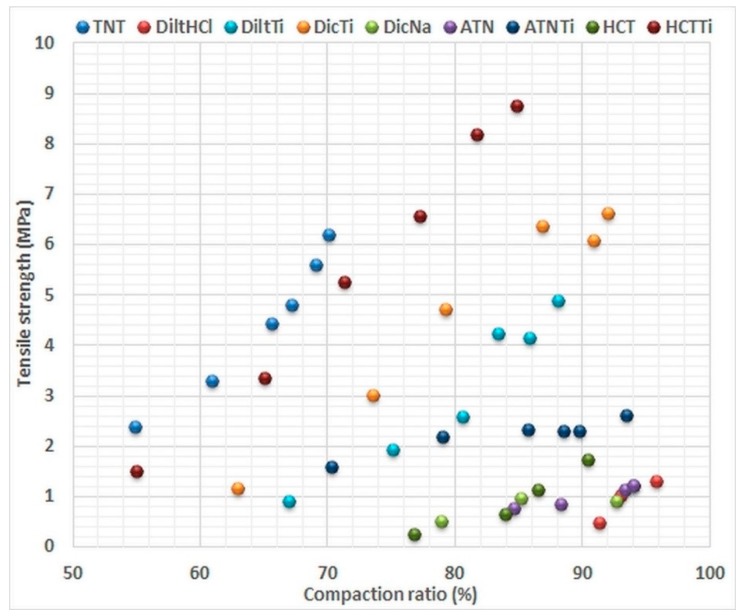
Evaluation of the tensile strength vs. the compaction ratio of the investigated materials compressed with 50, 100, 150, 200 and 250 MPa compression pressure.

The results indicate an opposing behaviour of the various APIs and the TNTs against compression. TNTs have poor compressibility (only 50% compaction ratio at low compression force) but have good compactibility (constantly increasing tensile strength and densification in the 50 to 250 MPa compression pressure range). In contrast, pure APIs have appropriate compressibility, as they show a high (approx. 70–90%) compaction ratio at low pressure, but poor compactibility, which may be seen from the slightly increasing compaction ratios and tensile strengths. Nevertheless, a highly improved compactibility may be established as result of the composite formation, providing 4 to 6 times higher tensile strength at same compaction ratio than that of the investigated APIs (Figure 8). On the other hand, despite similar tendencies, the incorporated APIs endowed the composites with unique characteristics. The highest improvement was observed in the case of DicTi where, in contrast with the pure API, no tensile strength limitation was noticed, due to the lack of lamination above 85% compaction ratio. DiltTi, where the API was also well incorporated into the composite, showed similar tablet properties (Figure 6). This indicates considerable predominance of TNTs in systems with successful composite formation.

An interesting phenomenon is that HCTTi, where a reverse mechanism of the composite formation was observed, not only showed far better tablet properties than the other composites, but was very close to the results of pure TNTs, which was probably the result of the positive consonance of the reverse interaction HCT and TNTs, and the good compactibility of both constituents. In contrast, ATNTi displayed relatively bad tablet properties, which proves the important deficiency of the composite formation, and the induced dominance of the unfavourable compactibility of ATN in the physical-mixture-like ATNTi product. Nevertheless, it is remarkable that the higher tensile strength induced by compression of the composites was associated with lower compaction ratios, which indicates the potential to prepare hard tablets with relatively high porosity, thus providing benefits for drug release.

### 4.4. Relation of the Compression Energies and Post-Compressional Properties

**Figure 9:**
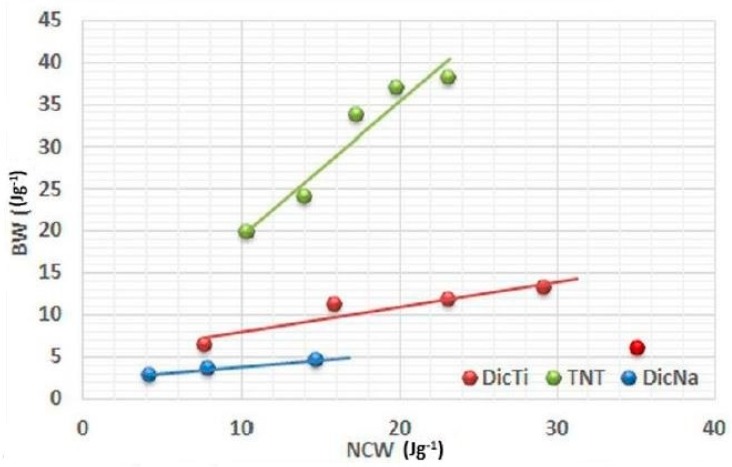
Evolution of breaking energy (BW) with NCW of TNT, DicNa and DicTi at 50, 100, 150, 200 and 250 MPa compression pressure.

The evolution of the breaking energy with the net energy allowed estimation of the efficacy of compaction through the efficacy coefficient, determined as the slope of the plotted (BW) = f(NCW) function (Figure 9). In addition, the C_eff_ values also permitted us to calculate the global yield R_T_ (Table 2), which presents the option to express the global ability of the powders to be transformed into cohesive compacts and describe the compaction profile of the materials in a single value, while leaving the particulars of the compression cycle out of account.

The best R_T_ value was shown by the TNTs, which supports expectations of their exceptional compressibility, and a considerable improvement in the global yield was observed in the case of all API–TNT composites in comparison with the pure API, despite the sometimes antinomic efficacy coefficient values. The only exception was ATN/ATNTi, wherein the inhomogeneous, physical-mixture-like API–TNT product was predominated by the characteristics of the ATN crystals.

In regard to the C_eff_, TNTs exhibited by far the highest values, thus they proved to have the best ability to convert net energy into cohesion among the tested materials, despite their relatively low compaction ratio. In contrast, for the pure APIs the bigger compaction ratio was correlated with higher C_eff_ value. This discrepancy may be the explanation for the unexpected results observed for composite forms. Namely, in the case of DicNa and HCT the composite formation resulted in higher C_eff_ values, while decreased efficacy coefficient was detected for the composite forms of DiltHCl and ATN in comparison with the pure API. This indicates that the C_eff_ is related to multiplication of the tensile strength increment rate and the minimal compaction ratio, which provides measurable tablet hardness. Therefore, it is noteworthy that the C_eff_ may hide important details, such as the absolute value of tablet hardness, and may show good results for products where the quality of the API tablets proved to be doubtful (e.g., laminated or mouldering) in many instances, which hindered the performance of certain breaking tests and necessitated the estimation of C_eff_ values from less data (seen for DicNa in Figure 7). Overall, it can be established that the C_eff_ value alone is not suitable for the estimation of powder compactibility, and R_T_ gives a more representative picture of the relationship between the compression energy and the post-compressional properties of tablets.

## 5. Conclusions

The present work aimed to investigate the effect of composite formation with TNTs on the compressibility and compactibility of incorporated APIs, and at the same time to reveal the relevance of the use of TNTs for tabletting nanocrystalline APIs.

In conclusion, the measurements revealed that TNTs are promising carrier materials, providing important technological benefits in the field of direct tablet compression. Based on the results, the use of TNTs has advantages in every step of tabletting. TNTs improved the flowability of the incorporated APIs, and displayed favourable particle rearrangement in the initial part of the tabletting process. Furthermore, the presence of TNTs decreased the elastic recovery of the APIs and resulted in good conversion of energy into plastic deformation. Due to the high feasibility of direct compression of the composites, not only did the range of compressibility of the APIs become larger through incorporation, but the post-compressional properties were also found to be more advantageous for the API–TNT tablets than for the API tablets.

It is remarkable that the presence of TNTs improved the compactibility of the APIs in all cases, even when the composite formation was incomplete and the product was inhomogeneous. This observation proves that the processability of TNTs is extremely good, and establishes the idea that the TNTs can potentially be used as carriers in the manufacturing of nanocrystalline APIs, and also as efficient and cheap excipients for any drug in direct tabletting.

Overall, it can be stated that hydrothermally synthesised TNTs are promising carrier materials that can not only be beneficially used for various biopharmaceutical purposes in medical therapy, but also for technological objectives in nanocrystalline drug formulation and manufacturing.

## Figures and Tables

**Table 1:** Packing coefficient of the materials.

Material	C_t_ (%)
TNT	7.81
DiltHCl	58.42
DiltTi	10.57
DicNa	59.64
DicTi	18.02
ATN	60.04
ATNTi	20.03
HCT	46.24
HCTTi	22.45

**Table 2:** Energetic parameters of titanate nanotubes (TNTs), active pharmaceutical ingredients (APIs) and TNT–API composite powders.

Material	R_1_ (%)	R_2_ (%)	R_3_ (%)	Ceff (%)	R_t_ (%)
TNT	88.65	16.32	82.87	0.314	0.0376
DiltHCl	22.44	30.44	76.26	0.216	0.0113
DiltTi	82.26	29.79	88.15	0.132	0.0285
DicNa	25.52	29.52	59.16	0.017	0.0008
DicTi	76.36	31.32	89.38	0.185	0.0395
ATN	26.38	10.18	45.50	0.131	0.0046
ATNTi	74.17	21.46	71.5	0.043	0.0049
HCT	34.3	32.45	80.49	0.093	0.0083
HCTTi	67.25	28.34	82.31	0.268	0.0420

**Table 3:** Post-compressional properties of the materials.

Material/Pressure (MPa)	Compaction Ratio (%)	Breaking Force (N)	SD	Tensile Strength (MPa)	SD
TNT/50	54.94	172.55	2.82	2.35	0.04
TNT/100	60.98	212.72	8.62	3.28	0.09
TNT/150	65.74	274.48	8.23	4.42	0.20
TNT/200	67.29	299.67	8.59	4.78	0.12
TNT/250	69.13	325.56	-	5.58	-
DiltHCl/50	91.37	23.49	1.12	0.47	0.03
DiltHCl/100	93.04	48.49	2.44	1.01	0.07
DiltHCl/150					
DiltHCl/200	95.84	60.62	5.98	1.29	0.13
DiltHCl/250					
DicNa/50	78.98	23.31	2.08	0.49	0.04
DicNa/100	85.30	42.11	3.86	0.95	0.09
DicNa/150					
DicNa/200	92.72	46.42	10.53	0.90	0.33
DicNa/250					
ATN/50	84.73	22.30	2.31	0.73	0.07
ATN/100	88.35	24.13	5.00	0.83	0.17
ATN/150					
ATN/200	93.48	30.87	4.39	1.12	0.15
ATN/250	94.08	32.38	1.74	1.19	0.06
HCT/50	76.85	16.32	1.02	0.24	0.01
HCT/100	84.08	38.64	1.15	0.64	0.02
HCT/150	86.63	63.44	2.05	1.11	0.02
HCT/200	90.47	85.55	9.03	1.71	0.02
HCT/250					
DiltTi/50	67.05	83.33	3.13	0.87	0.03
DiltTi/100	75.15	150.92	6.92	1.90	0.17
DiltTi/150	80.70	248.11	23.15	2.56	0.77
DiltTi/200	83.50	330.98	5.64	4.22	0.13
DiltTi/250	85.89	307.15	-	4.13	-
DicTi/50	63.06	95.61	8.09	1.14	0.10
DicTi/100	73.65	228.63	12.15	2.99	0.27
DicTi/150	79.33	331.09	2.54	4.70	0.37
DicTi/200	86.96	361.03	10.32	6.35	0.54
DicTi/250	90.94	352.93	-	6.07	-
ATNTi/50	70.41	106.01	1.47	1.56	0.02
ATNTi/100	79.06	131.07	6.02	2.16	0.10
ATNTi/150	85.84	129.17	5.97	2.31	0.11
ATNTi/200	88.65	127.86	9.74	2.28	0.13
ATNTi/250	89.83	120.82	-	2.27	-
HCTTi/50	55.10	107.03	3.00	1.50	0.04
HCTTi/100	65.09	214.19	2.93	3.35	0.25
HCTTi/150	71.35	289.66	8.08	5.24	0.15
HCTTi/200	77.30	334.61	23.93	6.56	0.47
HCTTi/250	81.85	393.72	-	8.17	-

## Supplementary Materials

The following are available online at http://www.mdpi.com/1996-1944/11/12/2582/s1.
